# A systematic review of substance use screening in outpatient behavioral health settings

**DOI:** 10.1186/s13722-023-00376-z

**Published:** 2023-03-26

**Authors:** Diana Woodward, Timothy E. Wilens, Meyer Glantz, Vinod Rao, Colin Burke, Amy M. Yule

**Affiliations:** 1 Department of Psychiatry, Massachusetts General Hospital, Harvard Medical School, Boston, MA 02114 USA; 2 Private Clinical Practice, Rockville, MD USA; 3 Department of Psychiatry, Boston Medical Center, 850 Harrison Avenue, Boston, MA 02118 USA

**Keywords:** Screening, Substance use, Substance use disorder, Behavioral health, Outpatient

## Abstract

**Objective:**

Despite the frequent comorbidity of substance use disorders (SUDs) and psychiatric disorders, it remains unclear if screening for substance use in behavioral health clinics is a common practice. The aim of this review is to examine what is known about systematic screening for substance use in outpatient behavioral health clinics.

**Methods:**

We conducted a PRISMA-based systematic literature search assessing substance use screening in outpatient adult and pediatric behavioral health settings in PubMed, Embase, and PsycINFO. Quantitative studies published in English before May 22, 2020 that reported the percentage of patients who completed screening were included.

**Results:**

Only eight articles met our inclusion and exclusion criteria. Reported prevalence of screening ranged from 48 to 100%, with half of the studies successfully screening more than 75% of their patient population. There were limited data on patient demographics for individuals who were and were not screened (e.g., gender, race) and screening practices (e.g., electronic versus paper/pencil administration).

**Conclusions:**

The results of this systematic review suggest that successful screening for substance use in behavioral health settings is possible, yet it remains unclear how frequently screening occurs. Given the high rates of comorbid SUD and psychopathology, future research is necessary regarding patient and clinic-level variables that may impact the successful implementation of substance use screening.

*Trial registry* A methodological protocol was registered with the PROSPERO systematic review protocol registry (ID: CRD42020188645).

**Supplementary Information:**

The online version contains supplementary material available at 10.1186/s13722-023-00376-z.

## Introduction

Substance use disorders (SUD) pose a substantial societal burden in the United States. In 2020 alone, an estimated 28.3 million people aged 12 or older met criteria for a past-year alcohol use disorder, while 18.4 million people aged 12 or older experienced a past-year illicit drug use disorder [1] Risky substance use and SUD are associated with substantial disability and mortality, with an estimated 480000 tobacco-related deaths and 95000 alcohol-related deaths annually in the United States [2, 3]. Of particular concern, drug-related overdose deaths have risen over the past years, increasing from 70,630 deaths in 2019 to 92000 deaths in 2020 [4, 5].

Prior research has established psychopathology as a significant risk factor for developing a SUD [6–9]. For example, individuals with depression are approximately 2 times more likely to develop a SUD, and those with attention deficit hyperactivity disorder exhibit a 2.3 times greater risk [10]. Furthermore, individuals with one or more psychiatric diagnoses experience greater SUD severity [11, 12]. The sequelae of co-occurring SUD and psychiatric disorders include increased odds of additional psychopathology [15], hospitalizations [16], suicide attempts [13, 17, 18], overdose [19–21], criminal behavior [22], and homelessness [23]. Additionally, adults with co-occurring disorders report overall lower quality of life [24] and lower social and occupational functioning [13, 25, 26].

Despite the imposed burden of comorbid SUD and psychopathology, in 2019, 51.4% of individuals in the United States with co-occurring disorders received no treatment, 38.7% received mental health treatment only, 7.8% received treatment for both mental health and SUD, and 1.9% received SUD treatment only (27). Given that many treatment-seeking individuals with co-occurring SUD and psychopathology obtain mental health treatment rather than substance use treatment, screening for substance use concerns in behavioral health settings is necessary to identify individuals at the greatest risk for maladaptive outcomes.

To this end, both the Substance Abuse and Mental Health Services Administration (SAMHSA) and the National Institute for Health and Clinical Guidance (NICE) have urged mental health providers to routinely administer patient self-report questionnaires to screen for substance use [28, 29]. Most efforts to integrate substance use screening into clinical care have focused on primary care settings [30–33]. As such, the success of substance use screening tools in other outpatient settings remains unclear. Because behavioral health clinics generally have both fewer ancillary supports to assist with screening compared to primary care, as well as high staff turnover rates [34, 35], research is needed on screening for substance use in these settings. Hence, we aim to summarize the extant literature on systematic screening for substance use in behavioral health, with a focus on the prevalence of screening within these clinics, characteristics of the screening tools used, and screening practices.

## Methods

A methodological protocol was registered with the PROSPERO systematic review protocol registry (ID: CRD42020188645).

### Search strategy

We conducted a search based upon Preferred Reporting Items for Systematic Reviews and Meta-Analyses (PRISMA) guidelines of peer-reviewed literature within the PubMed, Embase, and PsycINFO databases through May 22, 2020 with no restrictions for the start date. We examined both the prevalence and frequency of substance use screening in outpatient behavioral health clinics as well as the characteristics of the outpatient behavioral health clinics that screen for substance use. We searched each database using various combinations of search terms that can be found in the Additional file 1. Bibliographies of reviewed articles were also examined for additional studies to ensure that no relevant articles were omitted.

Inclusion criteria were quantitative studies examining substance use screening in outpatient adult and pediatric behavioral health clinics published in English. This included general psychiatric clinics, community mental health organizations, university counseling centers, and other specialty services. Studies were only included if they implemented systematic screening for substance use and reported the percentage of patients who completed the screener. Editorials, commentaries, opinion papers, chapters, and research studies that recruited participants to complete screening tools were excluded. Studies examining screening for substance use only in integrated behavioral health settings within primary care, emergency rooms, or inpatient settings were also excluded. If studies examined screening for substance use in behavioral health-only clinics alongside integrated behavioral health settings they were included if they stratified screening rates by clinic type.

### Selection of studies

Two reviewers independently screened the titles and abstracts of all papers. Any disagreements were resolved by consensus, and irrelevant titles were excluded. A record was kept of all irrelevant and duplicate articles. The full text of the remaining papers was reviewed by the two investigators and included/excluded. A third senior investigator reviewed all the included papers to confirm they met inclusion/exclusion criteria.

### Data extraction, analysis, and synthesis

Data were extracted from the quantitative studies by one reviewer and discussed with the senior reviewer. The following variables were extracted: Setting, sample size, percentage of patients screened, patient demographics, language of screening tool, screener administered, substances screened, date of study, frequency of screening, and method of screening (computer, paper, self-report, clinician report, etc.).

## Results

**Fig. 1:**
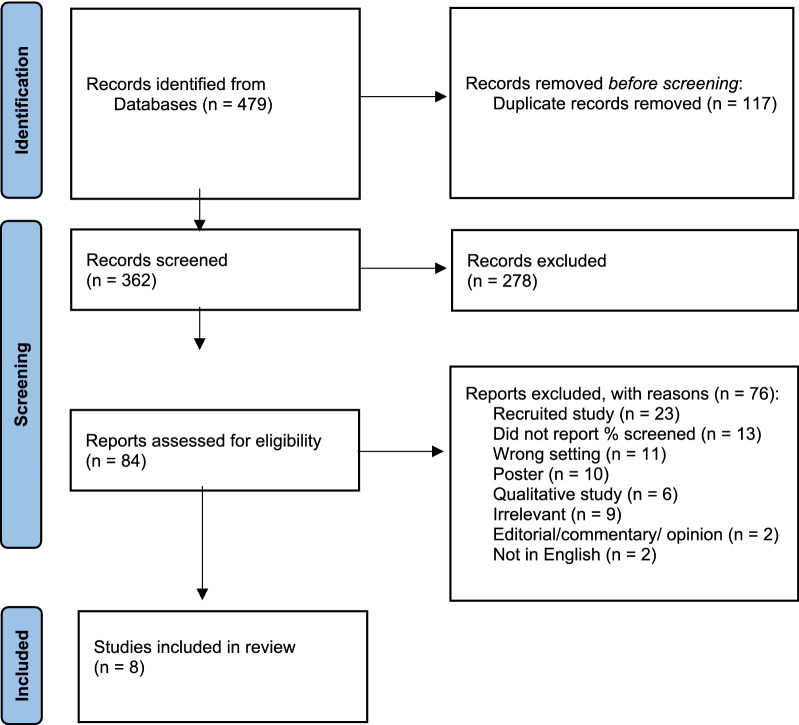
PRISMA diagram

Our initial search yielded 362 non-duplicate articles (Fig. 1). Eighty-four articles were determined to be potentially relevant and therefore reviewed in full. Of the 84 potentially relevant articles, 76 articles were excluded based on eligibility criteria (see Fig. 1). Eight articles were included in the final review (Table 1). The most common reasons for exclusion after full-text review were that the article reported on data from a sample recruited for a research study (N = 23), the authors did not report the percentage of patients screened (N = 13), or screening was implemented in a non-behavioral health setting (N = 11). The 8 articles included in this systematic review were published between 1992 and 2018. The sample sizes ranged from 88 to 22,956 screened patients.

**Table 1:** Studies reporting systematic screening for substance use in outpatient behavioral health settings

Author, Year	Setting description (number of settings, pediatric/adult)	Screener	Substances Screened	Frequency of Screening	Administration	Method of Screening	% Screened	N (screened)
Denering and Spear, 2012	University counseling center (1, adult)	AUDIT-C* (prescreen) ASSIST* (screen)	AUDIT-C*: Alcohol ASSIST*: Tobacco, alcohol, cannabis, cocaine, amphetamine, inhalants, sedatives, hallucinogens, opioids, other drugs	Intake	AUDIT-C*: self ASSIST*: clinician	Not Reported	100.0%	AUDIT-C*: 6,772 ASSIST*: 1534
Gabel, Radigan, Wang and Sederer, 2011	Outpatient and day treatment (48, pediatric)	CRAFFT Single cigarette use question Single drug use question	Cigarettes, alcohol, "street " drugs	CRAFFT: intake and 1 year Single cigarette and drug question: quarterly	Self	Not Reported	Smoking: 85.0% Alcohol/drug: 84.0%	2095
Karno, Granholmm and Lin, 2000	Outpatient Veterans Affairs Mental Health Clinic (1, adult)	AUDIT* CAGE*	Alcohol, "drug use"	Intake	Self	Paper	74.9%	197
King, Beehler, Wade, Buchholz, Funderburk, Lilienthal and Vair, 2018	Veterans Affairs outpatient and integrated care (Not Reported)	AUDIT-C* CAGE*	Alcohol	Intake	Not Reported	Not Reported	AUDIT-C: 80.4% CAGE: 00.0%	22,956 at least 1 screen
Ramo, Bahorik, Delucchi, Campbell and Satre, 2018	Outpatient and partial hospitalization (2, adult)	ASSIST*	Tobacco, alcohol, marijuana, prescription sedative/ hypnotic pills, cocaine, amphetamines, opioids, hallucinogens, ecstasy/ MDMA	Intake	Self	Computer	48.0%	405
Satre, Wolfe, Eisendrath and Weisner, 2008	Outpatient (1, adult)	Electronic Health Inventory If positive: SMAST*	Alcohol, cannabis, cocaine, amphetamines, hallucinogens, ecstasy, sedatives opioids, and tobacco	Intake	Self	Computer	75.0%	422
Silverman, O'Neill, Cleary, Barwick and Joseph, 1992	Outpatient (1, adult)	SMAST*	Alcohol	Intake	Self	Not Reported	88.0%	88
Stanhope, Manuel, Jessell and Halliday, 2018	Community health organizations (27, adult)	CRAFFT UNCOPE	Alcohol, marijuana, “anything else to get high”	Not Reported	Not Reported	Not Reported	93.5%	2873

* Screener abbreviations

*AUDIT* alcohol use disorders identification test; *CAGE* cut down, annoyed, guilty, eye opener; *ASSIST* alcohol, smoking and substance involvement screening test; *SMAST* short michigan alcoholism screening test

### Setting

Six of the eight studies were conducted in behavioral health clinics within a larger healthcare system, two of which took place in Veterans Affairs (VA) facilities [36–41]. The two studies that were not conducted in healthcare systems were conducted in a university counseling center [42] and community mental health organizations [43]. All studies were conducted in the United States. Four studies were single-site [37, 40–42], three studies included multiple sites ranging from 2 to 48 [36, 39, 43], and one study did not report the number of sites [38]. The majority of the studies (62.5%) were conducted in adult clinics [37, 39, 40, 42, 44], with one study focused on college students [42]. Two studies included pediatric patients [36, 43], and one study did not report age [41].

### Screener and substances screened

All of the studies screened for alcohol, the majority screened for drugs (N = 6) [36, 37, 39, 40, 42, 43], and half of the studies screened for tobacco (N = 4) [36, 39, 40, 42]. Of those that screened for drugs, two studies administered screeners which did not differentiate type of substance [36, 37]. Of the remaining four, all specifically queried about marijuana/cannabis [39, 40, 42, 43], and three screened for other drugs, including opioids [39, 40, 42]. A range of 1 to 5 screeners was used to assess for substance use. Additionally, one study administered both a pre-screening instrument and a screening instrument [42]. The most commonly used screeners were the Alcohol Use Disorders Identification Test-Concise (AUDIT-C) [38, 42, 45], the CRAFFT [36, 43, 46], the Alcohol, Smoking, and Substance Involvement Screening Test (ASSIST) [39, 42, 47], and the Short Michigan Alcohol Screening Test (SMAST) [40, 41, 48] (all N = 2).

### Frequency and methods of screening

The majority of studies reported screening only at intake (N = 6) [37–42]. One clinic implemented different screening instruments at intake, quarterly, and one year [36], and Stanhope et al. did not report the frequency of their screening across community mental health organizations. Of the eight studies, five relied solely on self-administration [36, 37, 39–41], one on both self- (prescreen) and clinician- (screen) administration [42], and two did not report how the screening was administered [38, 43]. Additionally, although the majority (N = 5) of authors did not report how information was collected [36, 38, 41–43], two studies utilized an electronic screen [39, 40] and one study relied on paper and pencil [37]. Finally, none of the studies reported the language of their screening instrument(s) [37].

### Screening rate

One study reported screening all patients [42]. The screening rates of the remaining studies ranged from 48 to 93.5% of patients. Screening in adult-only clinics ranged from 48 to 100% of patients [37–40, 42] while screening from clinics with adult and pediatric patients ranged from 84 to 93.5% [36, 43]. The screening rate using an electronic screen ranged from 48 to 75% of patients [39, 40], and the rate for paper/pencil was 74.9%.

### Demographics

#### Gender

Five studies reported on the gender of screened patients [36, 37, 39, 40, 43], and one study reported on gender across the total study population (patients who did and did not complete the screening) [38]. Of those that reported on the gender of screened patients, the range was 30 to 86% male. In two studies that did not report the gender across the total study population, the studies did report that there were no significant gender differences between patients who did and did not complete screening [40, 43].

#### Age

Four studies reported the mean age of screened patients, with a range of 16.6 to 42.9 years [37, 39, 40, 43]. Three studies reported mean age across the total study population, with a range of 36.1 to 53.5 years [38–40]. One additional study that did not report mean age across the total study population reported no significant difference in mean age between screened patients and the total study population [43].

#### Race

The three studies that reported on the race of screened patients included predominantly white patients, with these participants ranging from 52.8 to 72% of the sample [39, 40, 43]. The next most represented race was Asian, ranging from 9 to 10.5% of the sample. No studies reported race across the total study population; however, two studies reported no significant racial differences between patients who did and did not complete screening [40, 43].

#### Ethnicity

The two studies that reported on the ethnicity of screened patients included predominantly non-Hispanic patients, with these patients ranging from 73 to 93% of the sample [40, 43]. The one study that reported ethnicity across the total study population (patients who did and did not complete screening) was also largely non-Hispanic (94.2%) [38]. Two additional studies reported no significant differences in ethnicity between patients who did and did not complete screening; however, they did not report ethnicity type for the study population [40, 43].

#### Psychiatric comorbidities

Though two studies provided descriptive information on psychopathology, neither compared psychopathology between those who were and were not screened in the clinic [37, 38]. Karno et al. reported rates of depressive disorder (48%), anxiety disorder (15%), bipolar disorder (13%), and schizophrenia/ schizoaffective disorder (11%) in screened patients. King and colleagues found that 15.1% of all clinic patients had a trauma/ stressor-related disorder (including post-traumatic stress disorder), and 12.9% of all clinic patients had a mood disorder.

## Conclusions

Our aim in this review was to determine the prevalence and the characteristics of screening practices for substance use in outpatient behavioral health clinics. Though we identified only 8 studies that met review criteria, half of these studies reported screening more than 75% of their patient population [36, 41–43].

The screening rates in the identified studies are comparable to those reported in a recent examination of substance use screening in primary care settings, which found that 71.8% of eligible patients were screened after implementation efforts [49]. However, whether existing research on screening for substance use represents standard practice in all behavioral health clinics remains unclear given limited reporting on this practice. While the 2020 National Mental Health Services Survey (N-MHSS) reported that approximately 54% of the 4,941 surveyed outpatient mental health treatment facilitates offer screening for tobacco use, it did not specify whether this screening is systematic and routine and did not report on screening for non-nicotine substances (50). Furthermore, the intent to screen for substance use does not always translate into clinical practice. A large survey found that although 93.1% and 78.9% of mental health clinic directors reported having screening guidelines for alcohol and illicit substance use, respectively, only 66.6% and 57.8% of clinic staff reported conducting said screening [51].

Several patient- and clinic-level variables influence the successful implementation of systematic screening. Unfortunately, few studies in the current review reported patient demographic information. We were therefore unable to identify specific patient demographics associated with a high prevalence of screening for substance use or demographic differences between patients who were and were not screened to help identify patient groups who did not complete screening. This is notable since research from the primary care setting has found differences in screening for substance use based on demographics. For example, Black and Hispanic patients and adults over the age of 65 may require more assistance to complete electronic screening for substance use due to problems with comprehension or technical issues [52]. In light of increasing overdose deaths among Black and Hispanic youth [53], research examining the barriers to screening for substance use in particular demographic groups is needed to ensure equitable care.

Clinic factors that influence the successful implementation of screening center around the method of screening administration. For most studies in our review, screening tools were administered as patient self-report [36, 37, 39–41]. This is consistent with recent research in primary care and emergency department settings showing increased patient comfort with self-report screening compared to clinician-administered screening [54–56], particularly amongst individuals who belong to groups who are more stigmatized for substance use [52, 57, 58]. Another notable finding of our review was the omission of data regarding screening tools (paper and pencil versus electronic) and language of screening. A review of screening in primary care found that electronic questionnaires using patient self-report in both pediatric and adult settings improved data quality and completion time, decreased costs, and were preferred by patients. However, the use of electronic questionnaires also led to increased privacy concerns and access challenges [59]. Electronic measures, particularly those linked to the electronic medical record, may also result in racial and ethnic disparities in screening completion rates [60]. Additional research in the behavioral health setting is needed to determine patient and clinician preferences regarding the method of screening, particularly for more stigmatized conditions such as substance use [61, 62].

Finally, the timing and frequency of screening is another important factor to consider during implementation. Most studies in our systematic review reported screening patients for substance use only at intake [36–43]. Although screening at intake identifies patients who may benefit from SUD treatment [63], ongoing screening and progress monitoring improves engagement in SUD treatment and SUD outcomes [64, 65], and a recent consensus panel organized by SAMHSA recommended screening patients with psychiatric disorders for substance use annually [66]. Thus, future research should examine the prevalence and success of repeated screening for substance use.

The results of our review need to be considered in light of methodological limitations. The generalizability of the findings may be limited given the small number of eligible manuscripts. Moreover, several of these studies were missing information on patient- and clinic-level variables related to implementation that was recently identified as necessary to report on for studies evaluating the use of patient self-report questionnaires to improve the methodological quality, transparency, and applicability of the findings [67]. Hence it was difficult to conclude what variables contributed to the successful implementation of screening for substance use in the behavioral health setting. Furthermore, of those studies that did report patient demographics, the majority of the subjects were adults, white, and non-Hispanic. As such, the results may not be generalizable to pediatric or more diverse racial and ethnic groups. Additionally, to narrow the scope of the current review, we excluded manuscripts that examined substance use screening in integrated behavioral health clinics within primary care. Although implementation in these settings is important to investigate to better understand the overall landscape of screening for substance use in settings that provide behavioral health care, integrated behavioral health clinics likely face different barriers and facilitators. Lastly, more clinics may be systematically screening for substance use and not reporting their findings in published results. Thus, this topic is at risk for publication bias as behavioral health clinics that have struggled to implement systematic screening for substance use may not pursue publication.

In summary, the results of our review indicate that screening for substance use in the outpatient behavioral health setting can be successfully implemented at initial intake. Our review highlights the need for further examination of patient- and clinic-level variables that may impact the successful implementation of screening in behavioral health. Future research should include these variables to inform implementation efforts, ensure equity in screening, and achieve consistency with recent reporting guidelines [67].

## Supplementary Information

**Additional file 1**. Search terms used in the systematic review of substance use screening in outpatient behavioral health settings.

## Data Availability

All data generated or analyzed during this study are included in this published article [and its Additional information files].

## Abbreviations

SUD: Substance use disorder
SAMHSA: Substance Abuse and Mental Health Services Administration
NICE: National Institute for Health and Clinical Guidance
PRISMA: Preferred reporting items for systematic reviews and meta-analyses
VA: Veterans affairs
AUDIT: Alcohol use disorders identification test
CRAFFT: Car, relax, alone, forget, friends, trouble
ASSIST: Alcohol, smoking and substance involvement screening test
SMAST: Short Michigan alcohol screening test
N-MHSS: National Mental Health Services Survey

## References

[1] Substance Abuse and Mental Health Services Administration. Key Substance Use and Mental Health Indicators in the United States: Results from the 2020 National Survey on Drug Use and Health. 2020;156. https://www.samhsa.gov/data/sites/default/files/reports/rpt35325/NSDUHFFRPDFWHTMLFiles2020/2020NSDUHFFR1PDFW102121.pdf. Accessed 14 July 2022.

[2] Esser MB, Sherk A, Liu Y, Naimi TS, Stockwell T, Stahre M (2020). Deaths and years of potential life lost from excessive alcohol use—United States, 2011–2015. MMWR Morb Mortal Wkly Rep.

[3] National Center for Chronic Disease Prevention and Health Promotion (US) Office on Smoking and Health. The health consequences of smoking—50 years of progress: a report of the surgeon general. centers for disease control and prevention (US). 2014. https://www.ncbi.nlm.nih.gov/books/NBK179276/. Accessed 2 Aug. 2022. 24455788

[4] Ahmad FB, Cisewski JA, Rossen LM, Sutton P. Provisional drug overdose death counts national center for health statistics. 2022. https://www.cdc.gov/nchs/nvss/vsrr/drug-overdose-data.htm. Accessed 2 June 2022.

[5] Multiple Cause of Death 1999–2019 on CDC WONDER Online Database. Centers for disease control and prevention, national center for health statistics. 2020 https://www.cdc.gov/nchs/nvss/vsrr/drug-overdose-data.htm. Accessed 18 May 2021.

[6] Abraham HD, Fava M (1999). Order of onset of substance abuse and depression in a sample of depressed outpatients. Compr Psychiatry.

[7] Kessler RC (2004). The epidemiology of dual diagnosis. Biol Psychiatry.

[8] Merikangas KR, McClair VL (2012). Epidemiology of substance use disorders. Hum Genet.

[9] Wilens TE, Martelon M, Joshi G, Bateman C, Fried R, Petty C (2011). Does ADHD predict substance-use disorders? A 10-year follow-up study of young adults with ADHD. J Am Acad Child Adolesc Psychiatry.

[10] Groenman AP, Janssen TWP, Oosterlaan J (2017). Childhood psychiatric disorders as risk factor for subsequent substance abuse: a meta-analysis. J Am Acad Child Adolesc Psychiatry.

[11] Russell BS, Trudeau JJ, Leland AJ (2015). Social influence on adolescent polysubstance use: the escalation to opioid use. Subst Use Misuse.

[12] Shane PA, Jasiukaitis P, Green RS (2003). Treatment outcomes among adolescents with substance abuse problems: The relationship between comorbidities and post-treatment substance involvement. Eval Program Plann.

[13] Baker KD, Lubman DI, Cosgrave EM, Killackey EJ, Yuen HP, Hides L (2007). Impact of co-occurring substance use on 6 month outcomes for young people seeking mental health treatment. Aust N Z J Psychiatry.

[14] Tolliver BK, Anton RF (2015). Assessment and treatment of mood disorders in the context of substance abuse. Dialogues Clin Neurosci.

[15] Mitchell JD, Brown ES, Rush AJ (2007). Comorbid disorders in patients with bipolar disorder and concomitant substance dependence. J Affect Disord.

[16] Curran GM, Sullivan G, Williams K, Han X, Allee E, Kotrla KJ (2008). The association of psychiatric comorbidity and use of the emergency department among persons with substance use disorders: an observational cohort study. BMC Emerg Med.

[17] Appleby L, Shaw J, Amos T, McDonnell R, Harris C, McCann K (1999). Suicide within 12 months of contact with mental health services: national clinical survey. BMJ.

[18] Oquendo MA, Currier D, Liu SM, Hasin DS, Grant BF, Blanco C (2010). Increased risk for suicidal behavior in comorbid bipolar disorder and alcohol use disorders: results from the national epidemiologic survey on alcohol and related conditions (NESARC). J Clin Psychiatry.

[19] Bohnert AS, Ilgen MA, Ignacio RV, McCarthy JF, Valenstein M, Blow FC (2012). Risk of death from accidental overdose associated with psychiatric and substance use disorders. Am J Psychiatry.

[20] Park TW, Lin LA, Hosanagar A, Kogowski A, Paige K, Bohnert AS (2016). Understanding risk factors for opioid overdose in clinical populations to inform treatment and policy. J Addict Med.

[21] Yule AM, Carrellas NW, Fitzgerald M, McKowen JW, Nargiso JE, Bergman BG (2018). Risk factors for overdose in treatment-seeking youth with substance use disorders. J Clin Psychiatry..

[22] Wilton G, Stewart LA (2017). Outcomes of offenders with co-occurring substance use disorders and mental disorders. Psychiatr Serv.

[23] Gonzalez G, Rosenheck RA (2002). Outcomes and service use among homeless persons with serious mental illness and substance abuse. Psychiatr Serv.

[24] Saatcioglu O, Yapici A, Cakmak D (2008). Quality of life, depression and anxiety in alcohol dependence. Drug Alcohol Rev.

[25] Kronenberg LM, Slager-Visscher K, Goossens PJJ, van den Brink W, van Achterberg T (2014). Everyday life consequences of substance use in adult patients with a substance use disorder (SUD) and co-occurring attention deficit/hyperactivity disorder (ADHD) or autism spectrum disorder (ASD): a patient’s perspective. BMC Psychiatry.

[26] Olfson M, Shea S, Feder A, Fuentes M, Nomura Y, Gameroff M (2000). Prevalence of anxiety, depression, and substance use disorders in an urban general medicine practice. Arch Fam Med.

[27] Substance Use and Mental Health Services Administration. 2019 National survey of drug use and health (NSDUH) Releases: U.S. department of health and human services. 2019; https://www.samhsa.gov/data/release/2019-national-survey-drug-use-and-health-nsduh-releases. Accessed 2 Aug. 2022.

[28] National Collaborating Centre for Mental Health (UK). Common mental health disorders: identification and pathways to care. british psychological society (UK). 2011 NICE Clinical Guidelines, No. 123. https://www.ncbi.nlm.nih.gov/books/NBK92266/. Accessed 2 Aug. 2022. 22536621

[29] Substance Use and Mental Health Services Administration. white paper on the evidence supporting screening, brief intervention and referral to treatment (SBIRT). 2011. https://www.samhsa.gov/sites/default/files/sbirtwhitepaper_0.pdf. Accessed 2 Aug. 2022.

[30] McNeely J, Kumar PC, Rieckmann T, Sedlander E, Farkas S, Chollak C (2018). Barriers and facilitators affecting the implementation of substance use screening in primary care clinics: a qualitative study of patients, providers, and staff. Addict Sci Clin Pract.

[31] McPherson TL, Hersch RK (2000). Brief substance use screening instruments for primary care settings: a review. J Subst Abuse Treat.

[32] Pilowsky DJ, Wu L-T (2012). Screening for alcohol and drug use disorders among adults in primary care: a review. Subst Abuse Rehabil.

[33] Rahm AK, Boggs JM, Martin C, Price DW, Beck A, Backer TE (2015). Facilitators and barriers to implementing screening, brief intervention, and referral to treatment (SBIRT) in primary care in integrated health care settings. Subst Abus.

[34] Paris M, Hoge MA (2010). Burnout in the mental health workforce: a review. J Behav Health Serv Res.

[35] Woltmann EM, Whitley R, McHugo GJ, Brunette M, Torrey WC, Coots L (2008). The role of staff turnover in the implementation of evidence-based practices in mental health care. Psychiatr Serv.

[36] Gabel S, Radigan M, Wang R, Sederer LI (2011). Health monitoring and promotion among youths with psychiatric disorders: program development and initial findings. Psychiatr Serv.

[37] Karno M, Granholm E, Lin A (2000). Factor structure of the alcohol use disorders identification test (audit) in a mental health clinic sample. J Stud Alcohol.

[38] King PR, Beehler GP, Wade M, Buchholz LJ, Funderburk JS, Lilienthal KR, et al. 2018. Opportunities to improve measurement based care practices in mental health care systems a case example of electronic mental health screening and measurement. Fam Syst Health. 10.1037/fsh0000379 10.1037/fsh0000379 30589320

[39] Ramo DE, Bahorik AL, Delucchi KL, Campbell CI, Satre DD (2018). Alcohol and drug use, pain and psychiatric symptoms among adults seeking outpatient psychiatric treatment: latent class patterns and relationship to health status. J Psychoactive Drugs.

[40] Satre D, Wolfe W, Eisendrath S, Weisner C (2008). Computerized screening for alcohol and drug use among adults seeking outpatient psychiatric services. Psychiatr Serv.

[41] Silverman DC, O'Neill SF, Cleary PD, Barwick C, Joseph R (1992). Recognition of alcohol abuse in psychiatric outpatients and its effect on treatment. Psychiatr Serv.

[42] Denering LL, Spear SE (2012). Routine use of screening and brief intervention for college students in a university counseling center. J Psychoactive Drugs.

[43] Stanhope V, Manuel JI, Jessell L, Halliday TM (2018). Implementing SBIRT for adolescents within community mental health organizations: a mixed methods study. J Subst Abuse Treat.

[44] King WM, Restar A, Operario D (2021). Exploring multiple forms of intimate partner violence in a gender and racially/ethnically diverse sample of transgender adults. J Interpers Violence.

[45] Bush K, Kivlahan DR, McDonell MB, Fihn SD, Bradley KA, Project ACQI (1998). The AUDIT alcohol consumption questions (AUDIT-C) an effective brief screening test for problem drinking. Arch Intern Med.

[46] Knight JR, Sherritt L, Shrier LA, Harris SK, Chang G (2002). Validity of the CRAFFT substance abuse screening test among adolescent clinic patients. Arch Pediatr Adolesc Med.

[47] WHO Assist Working Group (2002). The alcohol, smoking and substance involvement screening test (ASSIST): development, reliability and feasibility. Addiction.

[48] Selzer ML, Vinokur A, van Rooijen L (1975). A self-administered short Michigan alcoholism screening test (SMAST). J Stud Alcohol.

[49] McNeely J, Adam A, Rotrosen J, Wakeman SE, Wilens TE, Kannry J (2021). Comparison of methods for alcohol and drug screening in primary care clinics. JAMA Netw Open..

[50] Substance Abuse and Mental Health Services Administration. National Mental Health Services Survey (N-MHSS) 2020 data on mental health treatment facilities. 2020. https://www.samhsa.gov/data/report/national-mental-health-services-survey-n-mhss-2020-data-mental-health-treatment-facilities. Accessed 2 Aug. 2022.

[51] Sundström C, Petersén E, Sinadinovic K, Gustafsson P, Berman AH (2019). Identification and management of alcohol use and illicit substance use in outpatient psychiatric clinics in Sweden: a national survey of clinic directors and staff. Addict Sci Clin Pract.

[52] Adam A, Schwartz RP, Wu L-T, Subramaniam G, Laska E, Sharma G (2019). Electronic self-administered screening for substance use in adult primary care patients: feasibility and acceptability of the tobacco, alcohol, prescription medication, and other substance use (myTAPS) screening tool. Addict Sci Clin Pract.

[53] Spencer M, Warner M, Bastian BA, Trinidad JP, Hedegaard H (2019). Drug overdose deaths involving fentanyl, 2011–2016. Natl Vital Stat Rep.

[54] Chisolm DJ, Gardner W, Julian T, Kelleher KJ (2008). Adolescent satisfaction with computer-assisted behavioural risk screening in primary care. Child Adolesc Ment Health.

[55] Paperny DM, Aono JY, Lehman RM, Hammar SL, Risser J (1990). Computer-assisted detection and intervention in adolescent high-risk health behaviors. J Pediatr.

[56] Rhodes KV, Lauderdale DS, Stocking CB, Howes DS, Roizen MF, Levinson W (2001). Better health while you wait: a controlled trial of a computer-based intervention for screening and health promotion in the emergency department. Ann Emerg Med.

[57] Jimenez DE, Bartels SJ, Cardenas V, Alegría M (2013). Stigmatizing attitudes toward mental illness among racial/ethnic older adults in primary care. Int J Geriatr Psychiatry.

[58] Small J, Curran GM, Booth B (2010). Barriers and facilitators for alcohol treatment for women: are there more or less for rural women?. J Subst Abuse Treat.

[59] Meirte J, Hellemans N, Anthonissen M, Denteneer L, Maertens K, Moortgat P (2020). Benefits and disadvantages of electronic patient-reported outcome measures: systematic review. JMIR Perioper Med..

[60] Sisodia RC, Rodriguez JA, Sequist TD (2021). Digital disparities: lessons learned from a patient reported outcomes program during the COVID-19 pandemic. JAMIA Open.

[61] Earnshaw VA, Bogart LM, Menino DD, Kelly JF, Chaudoir SR, Reed NM (2019). Disclosure, stigma, and social support among young people receiving treatment for substance use disorders and their caregivers: a qualitative analysis. Int J Ment Health Addict.

[62] Kelly JF, Greene MC, Abry A (2021). A US national randomized study to guide how best to reduce stigma when describing drug-related impairment in practice and policy. Addiction.

[63] Simon KM, Harris SK, Shrier LA, Bukstein OG (2020). Measurement-based care in the treatment of adolescents with substance use disorders. Child Adolesc Psychiatr Clin N Am.

[64] Fadus MC, Squeglia LM, Valadez EA, Tomko RL, Bryant BE, Gray KM (2019). Adolescent substance use disorder treatment: an update on evidence-based strategies. Curr Psychiatry Rep.

[65] Van Horn DHA, Goodman J, Lynch KG, Bonn-Miller MO, Thomas T, Del Re AC (2020). The predictive validity of the progress assessment, a clinician administered instrument for use in measurement-based care for substance use disorders. Psychiatry Res.

[66] Substance Abuse and Mental Health Services Administration. TIP 42: Substance use disorder treatment for people with co-occurring disorders. 2020; 42. https://store.samhsa.gov/product/tip-42-substance-use-treatment-persons-co-occurring-disorders/PEP20-02-01-004. Accessed 28 July 2022. 34106563

[67] Gagnier JJ, Lai J, Mokkink LB, Terwee CB (2021). COSMIN reporting guideline for studies on measurement properties of patient-reported outcome measures. Qual Life Res.

