# Supplementary material for: A systematic review of substance use screening in outpatient behavioral health settings
Source: Addict Sci Clin Pract. 2023 Mar 26;18:18. doi: 10.1186/s13722-023-00376-z (PMC10041696; doi:10.1186/s13722-023-00376-z)
Supplement: Supplementary file 1 — Additional file 1. Search terms used in the systematic review of substance use screening in outpatient behavioral health settings. [file 13722_2023_376_MOESM1_ESM.docx]

Additional file: Search terms used in the systematic review of substance use screening in outpatient behavioral health settings.

Start Date: N/A, no limits placed on start date

Date searched (end date): 5/10/2020

| **Database: PubMed** | **Search words:** | **Limits (filter, limits, refine)** | **Records** |
| --- | --- | --- | --- |
| #1 | Behavioral health clinic*[tiab] OR  Community mental health clinic*[tiab] OR  "Community Mental Health Services"[Mesh] OR  "Community Mental Health Centers"[Mesh] OR  ("Inpatients"[Mesh] AND “Psychiatry”[Mesh]) OR  “Outpatient psychiatry”[tiab] OR  “Psychiatry outpatient”[tiab] OR  “Outpatient mental health”[tiab] OR  “Mental health clinic”[tiab] OR  “MH services”[tiab] OR  “outpatient psychiatric”[tiab] OR  “Emergency Psychiatry”[tiab] OR  “Community mental health service”[tiab] OR  “Mental health department”[tiab] OR  “youth mental health care”[tiab] OR  Psychiatric program*[tiab] | None |  |
| #2 | "Mass Screening"[Mesh] OR  Screen*[tiab] OR  Computerized screening[tw] OR  Computerized screening[tiab] OR  substance use questionnaire[tiab] OR  intake assessment*[tiab] OR  standardized questionnaire*[tiab] OR  standardized forms[tiab] | None |  |
| #3 | "Substance-Related Disorders"[mesh] OR  “Drug addiction”[tiab] OR  “Addiction”[tiab] OR  “Drug dependency”[tiab] OR  “Alcoholism”[mesh] OR  “Hazardous drinking”[tiab] OR  “Cannabis”[mesh] OR  “Alcohol”[tiab] OR  “Alcohol Drinking”[mesh] OR  “Alcoholic Intoxication”[mesh] OR  “Alcoholism”[mesh] OR  “Marijuana Abuse”[mesh] OR  “Binge Drinking”[mesh] OR  “Marijuana”[tiab] OR  “Marijuana Smoking”[mesh] OR  “Smoking Cessation”[mesh] OR  “Tobacco”[mesh] OR  drug abuse[tiab] OR  Alcohol abuse[tiab] OR  SUD[tiab] | None |  |
| #4 **(final search strategy)** | #1 AND #2 AND #3 | None | 175 |

| **Database: Embase** | **Search words:** | **Limits (filter, limits, refine)** | **Records** |
| --- | --- | --- | --- |
| #1 | ‘Behavioral health clinic*’:ti,ab,kw OR  ‘Community mental health clinic*’:ti,ab,kw OR  'Community Mental Health Service'/exp OR  'Community Mental Health Center'/exp OR  ‘Outpatient psychiatry’:ti,ab,kw OR  ‘Psychiatry outpatient’:ti,ab,kw OR  ‘Outpatient mental health’:ti,ab,kw OR  ‘Mental health clinic’:ti,ab,kw OR  ‘Mental health center’/exp OR  ‘MH services’:ti,ab,kw OR  ‘outpatient psychiatric’:ti,ab,kw OR  'Inpatient Psychiatry':ti,ab,kw OR  ‘Emergency Psychiatry’:ti,ab,kw OR  ‘emergency psychiatry’/exp OR  ‘Community mental health service’:ti,ab,kw OR  ‘Mental health department’:ti,ab,kw OR  ‘youth mental health care’:ti,ab,kw OR  ‘Psychiatric program*’:ti,ab,kw | None |  |
| #2 | 'Mass Screening'/de OR  ‘drug screening’/de OR  ‘screening’/de OR  Screen*:ti,ab,kw OR  ‘Computerized screening’:ti,ab,kw OR  ‘Computerized screening’:ti,ab,kw OR  ‘substance use questionnaire’:ti,ab,kw OR  ‘intake assessment*’:ti,ab,kw OR  ‘standardized questionnaire*’:ti,ab,kw OR  ‘standardized forms’:ti,ab,kw OR  ‘Questionaire’/exp OR  ‘assessment’/exp OR  ‘health assessment questionnaire’/exp | None |  |
| #3 | 'Substance?Related Disorders':ti,ab,kw OR  ‘substance use’/exp OR  ‘drug dependence’/exp OR  ‘Drug addiction’:ti,ab,kw OR  ‘Addiction’:ti,ab,kw OR  ‘Drug dependency’:ti,ab,kw OR  'Alcoholism'/de OR  ‘Hazardous drinking’:ti,ab,kw OR  ‘alcohol abuse’/exp OR  ‘drug abuse’/exp OR  'Cannabis'/exp OR  ‘Alcohol’:ti,ab,kw OR  'Alcohol Drinking':ti,ab,kw OR  ‘heavy drinking’/exp OR  'Alcoholic Intoxication'/exp OR  ‘cannabis addiction’/exp OR  'Marijuana Abuse':ti,ab,kw OR  'Binge Drinking'/exp OR  Marijuana:ti,ab,kw OR  ‘cannabis’:ti,ab,kw OR  ‘cannabis Smoking'/exp OR  ‘marijuana smoking’:ti,ab,kw OR  'Smoking Cessation'/exp OR  'Tobacco'/exp OR  ‘drug abuse’:ti,ab,kw OR  ‘Alcohol abuse’:ti,ab,kw OR  ‘SUD’:ti,ab,kw | None |  |
| #4 **(final search strategy)** | #1 AND #2 AND #3 | None | 207 |

| **Database: PsycINFO** | **Search words:** | **Limits (filter, limits, refine)** | **Records** |
| --- | --- | --- | --- |
| #1 | Behavioral health clinic*.ti,ab. OR  Community mental health clinic*.ti,ab. OR  exp Community Mental Health Services/ OR  exp Community Mental Health Centers/ OR  Inpatients Psychiatry:ti,ab,kw  Outpatient psychiatry.ti,ab. OR  Psychiatry outpatient.ti,ab. OR  Outpatient mental health.ti,ab. OR  Mental health clinic.ti,ab. OR  MH services.ti,ab. OR  outpatient psychiatric.ti,ab. OR  Emergency Psychiatry.ti,ab. OR  Community mental health service.ti,ab. OR  Mental health department.ti,ab. OR  youth mental health care.ti,ab. OR  Psychiatric program*.ti,ab. | None |  |
| #2 | Health Screening/ OR  Exp screening tests/ OR  Exp Drug usage screening/ OR  Screen*.ti,ab. OR  Computerized screening.mp. OR  Computerized screening.ti,ab. OR  Exp computerized assessment/ OR  intake assessment*.ti,ab OR  Exp intake interview/ OR  standardized questionnaire*.ti,ab OR  standardized forms.ti,ab OR  “substance use” questionnaire.ti,ab | None |  |
| #3 | Substance?Related Disorders:ti,ab OR  Exp Substance related and addictive disorders/ OR  Drug addiction.ti,ab. OR  Addiction.ti,ab. OR  Drug dependency.ti,ab. OR  Hazardous drinking.ti,ab. OR  exp Cannabis/ OR  Alcohol.ti,ab. OR  Marijuana.ti,ab. OR  exp drugs/ OR  exp Smoking Cessation/ OR  drug abuse.ti,ab. OR  Alcohol abuse.ti,ab. OR  SUD.ti,ab. | None |  |
| #4 **(final search strategy)** | #1 AND #2 AND #3 | None | 97 |
